# ceRNAs in Cancer: Mechanism and Functions in a Comprehensive Regulatory Network

**DOI:** 10.1155/2021/4279039

**Published:** 2021-10-07

**Authors:** Ni Yang, Kuo Liu, Mengxuan Yang, Xiang Gao

**Affiliations:** Second Hospital of Hebei Medical University, Shijiazhuang 050000, China

## Abstract

Noncoding RNAs have been shown with powerful ability in post-transcriptional regulation, enabling intertwined RNA crosstalk and global molecular interaction in a large amount of dysfunctional conditions including cancer. Competing endogenous RNAs (ceRNAs) are those competitively binding with shared microRNAs (miRNAs), freeing their counterparts from miRNA-induced degradation, thus actively influencing and connecting with each other. Constantly updated analytical approaches boost outstanding advancement achieved in this burgeoning hotspot in multilayered intracellular communication, providing new insights into pathogenesis and clinical treatment. Here, we summarize the mechanisms and correlated factors under this RNA interplay and deregulated transcription profile in neoplasm and tumor progression, underscoring the great significance of ceRNAs for diagnostic values, monitoring biomarkers, and prognosis evaluation in cancer.

## 1. Pervasive Noncoding RNAs in Genomic Scope

Numerous evidence has emerged regarding the noncoding properties of RNA transcripts over the past years, unraveling whose great capacity that goes far beyond the previously well-characterized genetic information carrier and indispensable messenger for protein synthesis to post-transcriptional regulation and multilayered sequence interactions, whereby extensively interweaved molecular crosstalk along with rapidly changing cellular environment composes a robust intracellular connection. The identified verification and importance of prevalent noncoding RNAs (ncRNAs) in evolutionary complexity of diverse organisms lies partially in the comparison, where up to 75%–95% of the human genome is transformed into RNA transcripts basically varying in length and functions, with actually less than 2%, quantitively 21,000 genes [1], attributed with protein-coding properties [2, 3], yet almost genome-wide translation has been confirmed in simply structured species such as unicellular yeast [4], and Caenorhabditis elegans possess about an equal amount of genes encoding a protein with human but a 30 times smaller genome [5]. Mingled with multiple contributing factors, various modes of transcription generate products including but not limited to antisense strands or noncoding intergenic transcripts, which were previously unrecognized and thought to be useless remainders of an immature expressing mechanism. Taking 200 base pairs as a boundary, noncoding transcripts are roughly divided into two categories corresponding with their size, namely small ncRNAs and long ncRNAs (lncRNAs) [6]. Small ncRNAs have been deeply functionalized, among which miRNAs enrolled in the intricate RNA-RNA regulation network are one of the most representative components and will be clarified later in detail, concerning their noteworthy roles in biochemical behaviors and pathophysiological conditions [3, 7, 8]. Conversely, presenting pleiotropic effects as guides, scaffolds, natural decoys, and sponges in large-scale molecular correlations in transcriptional, post-transcriptional, epigenetic, and gene-expressing events, lncRNAs have been reportedly viewed as “master regulator” [9], even so, those hitherto hidden approaches through which lncRNAs flexibly participate in cellular homeostasis stabilization and response to perturbation in development of plenty of diseases, herein exemplified by cancer, still await further exploration. Based on existing achievements underscoring the remarkable potential of ncRNAs as regulating elements in carcinogenesis, we overview the profile of ncRNAs, their derived identity as ceRNAs in tumor pathogenesis, and objective conditions affecting their inner action, which facilitates the renewal of highly targeted predicting tools and lays a deeply rooted foundation for future research and clinical implications in biomarker detection, prognosis judgement, and therapeutic regimen selection.

## 2. ceRNA Hypothesis: Derivation and Extension

Transcribed mainly from introns of coding genes, with the rest from exons of coding genes as well as intronic and exonic regions of noncoding sequences [10], miRNAs are small single-stranded RNAs consisting of generally 19–23 nucleotides, centralizing ceRNAs to regulate and interplay with each other by recognizing miRNA response elements (MREs) of target transcripts [2, 11, 12]. miRNA biosynthesis is a sequential enzyme-dependent process, in which canonically transcribed precursor miRNAs (pre-miRNAs) in the nucleus, in tandem catalyzed by RNA polymerase and nuclear Drosha/DGCR8 complex, are released into the cytoplasm and cleaved into double-stranded miRNAs with appropriate length by Dice, and finally incorporated into Argonaute-loaded miRNA-induced silencing complexes (miRISC) after degeneration of complementary strands [6, 13], base pairing with targets under the direction of 6–8 nucleotides in miRNAs' 5′ ends [9]. Seed matches in target transcripts are required for binding with miRNAs, while perfect complementarity is not always necessary. MREs are commonly 2–8 nucleotides sited in coding sequences (CDS), 5′ untranslated regions (5′ UTRs), and mostly 3′ UTRs of several subsets of RNAs comprising lncRNAs, transcribed pseudogenes, circular RNAs (circRNAs), and mRNAs [14–16], which are subjected to inhibition on expression through either complete degradation or translation repression, respectively, occurring when in high or relatively low degrees complementary base pairs are matched between MREs and miRNAs [2, 3]. Some have pointed out that imperfect bindings, implying “bulged sponges” in seed regions, are more effective in soaking up miRNAs and serving as competing molecules partly because of the longer period of occupation; otherwise, miRNAs are released once the perfectly paired targets go through degradation [17]. As a mutual interaction, the availability and activity of miRNAs are impaired by their binding targets, some of which degrade them, while others sequester them from alternative sequences of interest [11, 18]. What's more, 3′ end modification of miRNAs and target RNA function in a mathematical titration principle also account for such reduction in miRNA levels [19]. Given the above, it is conceivable that miRNAs may act as axis center in complicated intracellular crosstalk in both homeostatic status and disturbed physiological milieux like cancer.

Theoretically, when various transcripts are targeted by the same miRNAs, an elevated transcription level of one side would alleviate miRNA-induced suppression on the other, leading to direct or indirect regulation on gene expression. Transfected into viral vectors, artificial sponges of specific miRNAs were exploited before natural targets came into view, which were transcribed by strong promoters and synthesized to bear repeated binding sites for aimed miRNAs, thus exhibiting fascinating effects on derepressing counterpart targets [20, 21], showing profound significance for RNA crosstalk, and more precisely, the formation of ceRNA hypothesis.

The first discovered natural sponge was lncRNA IPS1 found in *Arabidopsis thaliana*, which was observed to decoy phosphate starvation-induced miR-399 and subsequently help maintain the stability and abundance of its partner target PHO2 [22]. Unlike precedently clarified perfect complementarity in plants, the mismatched loop on the miRNA cleavage site made IPS1 bypass the impairment and competent for efficient binding [3, 22]. Following the uncovery of this phenomenon termed “target mimicry” [22], the parallel finding was disclosed in animal cells, where ectopic overexpression of MREs resulted in moderately declining miRNA levels and 1.5–2.5-fold accumulation of the targets [21]. Later in 2010, Herpesvirus saimiri transformed T cells were reported to express ncRNA H. saimiri U-rich RNAs (HSURs), which were correlated with miR-27 degradation and increased FOXO1 levels [23]. The underlying implication and mechanism of these promising discoveries were extended to the field of cancer when pseudogene PTENP1 was proved to share common miRNAs with its homologous coding RNA [24]. With the antecedent supporting evidence assembled, the ceRNA hypothesis was put forward in 2011, demonstrating that each miRNA has manifold RNA targets and most RNAs bear a wealth of MREs, thus endogenous coding and noncoding RNAs regulate and crosstalk with each other by competitively binding to the shared but limited miRNA pools [12]. In the same year, other three research reinforced the crucial role of ceRNAs in the molecular characterization of cancer cells [25–27].

Grouping all the noncoding RNAs and noncoding properties of mRNAs into a functional complexity, the ceRNA hypothesis essentially opens the window for a multilevel and trans-regulatory ceRNA network (ceRNET) over the transcriptome, where competition and interplay among all subsets of ceRNAs occur in direct, indirect, or secondary manners with the help of miRNAs, together shedding light on the biochemical mechanism and post-transcriptional-layered explanations for pathogenesis and progression of massive disordered conditions such as cancer. Moreover, correlation with other factors such as RBPs and transcription factors also influences ceRNAs' biological activities [28], and miRNAs similarly vie for potent binding with shared target pools [29]. Some suggested ceRNA concept to be expanded to whatever RNA crosstalk surrounding common regulators [6], while others by the same token proposed “ceRNome” as a notion referring to the integration of reciprocally tying RNA molecules in a comprehensive cellular environment [9], indicating that ceRNA crosstalk is in no way standalone but in a global post-transcriptional context.

## 3. Building Blocks of ceRNA

### 3.1. Pseudogenes

Previously regarded as nonfunctional relicts of their ancestral genes due to detrimental mutations impeding them from being translated into explicit phenotypes [2], pseudogenes are gradually performing as bona fide competitors for their cognate genes with highly homologous MRE overlaps. Independent epigenetic modifications in pseudogenes signify initiative and stable evolutionary conservation [2]. About 14,505 pseudogenes contribute to making up human genome according to GENCODE Release (version 24) [2], consisting of unprocessed pseudogenes originating from gene duplication, processed pseudogenes through reverse transcription, and de novo synthesized unitary pseudogenes with no coding partners [30], whose transcripts participate in gene regulation as antisense sequences or compelling miRNA decoys as a subset of lncRNAs in ubiquitously expressing and tumor-specific patterns [31].

### 3.2. lncRNAs

There are estimated approximately 17,910 lnRNAs varying in length from 0.2 to 100 kilobases [32], which display tissue and developmental complexity, align with functional and spatial diversity of chromatin modification, RNA processing in the nucleus, and gene coding management in cytoplasmic parts [33], counteracting the reportedly low abundance as competitive candidates for miRNA binding in given conditions [3]. More precisely, lncRNA X-inactive specific transcript (Xist) could act in cis to devitalize the entire chromosome [34], and HOX transcript antisense RNA (HOTAIR) function in trans to drive metastasis through gene expression regulation [35], and chromatin structure is remodeled by alternative splicing associated with metastasis-associated lung adenocarcinoma transcript 1 (MALAT1) [36]. The most studied lncRNA in hepatocellular carcinoma, highly upregulated in liver cancer (HULC), is able to disengage protein kinase catalytic *β* (PRKACB) from miR-372 restraint, therefore promoting cAMP response element-binding protein (CREB) phosphorylation and, in turn, amplifying HULC upregulation [37]. Besides, lncRNAs are of great importance in controlling cell differentiation and pluripotency maintenance with respect to the effects of long intergenic noncoding RNAs (lincRNAs) [38].

### 3.3. circRNAs

circRNAs are fairly abundant in mammalian cells, generated from nearly 20% of functional genes [39]. Self-circularization depends on covalent conjunction of 3′ and 5′ ends after “backsplicing,” conferring high stability to these loop RNA structures compared with their linear counterparts, due to lack of free terminals and thereby resistance to exonuclease-induced degradation and miRNA-mediated repression [40]. circRNA ciRS-7 was identified to contain 60–70 MREs for miR-7 [40], acting as crucial regulators in cerebral development and tumorigenesis [40, 41]. Considerable evidence disclosed key roles of circRNAs as ceRNAs with dominant intracellular localization in malignancy progression [42], and the distinct stability makes them ideal biomarkers in body fluids such as blood or saliva for clinical assessment [43, 44].

### 3.4. mRNAs

Since more than 60% of human mRNAs harbor MREs according to computational prediction [45], it is unsurprising to postulate that the function of protein-coding RNAs is no more restricted to translation templates but propagated to active fine-tuners in ncRNA-mRNA and mRNA-mRNA crosstalk, which may give rise to accordant or opposite effects with their inherent encoding features. It has been widely studied that VAPA, CNOT6L, ZEB2, and VCAN mRNAs are ceRNAs for tumor suppressor PTEN mRNA, representing aberrant transcription levels and resulting in downregulation of PTEN mRNAs in a Dicer-dependent way in various cancer types such as colorectal cancer [25], breast carcinoma [46], and melanoma [26]. Similarly, other classically identified molecules include VCAN and CD44 with their competing RNAs, endowed with complex roles in cell proliferation, invasive behaviors, and some other malignant signatures in contexts of cancer [47, 48].

## 4. ceRNA Crosstalk Decipherment

Increasing computational, mathematical, and experimental tools have been posed for decoding ceRNA crosstalk and identifying putative candidates for their topology and dynamic fluctuation. Typical verifying process of ceRNA interactions successively includes corroborative tests such as RNA immunoprecipitation for miRNA-ceRNA binding, confirmation of positive correlation of transcription levels of ceRNAs, repeated miRNA-dependency tests through Dicer knock-out or MRE mutations, and finally epigenetic changes induced by up- or downregulation of ceRNAs in disrupted physiological conditions [6]. Prediction algorithms including PITA, TargetScan, miRanda, and rna22 have been validated efficient for seeking ceRNAs through recognition of MREs and scoring overlaps in quantified assessment, forming the database of predicted ceRNA interactions (ceRDB), yet the unclear targeting rules and incomplete complementarity brought out limitations in some cases [49]. With high-throughput sequencing of RNA isolated by crosslinking immunoprecipitation (HITS-CLIP) and photoactivatable ribonucleoside-enhanced crosslinking and immunoprecipitation (PAR-CLIP) introduced into wide use, RISC-bound targets are more efficiently and precisely identified [49–51]. Moreover, MS2-tagged RNA affinity purification (MS2-TRAP) makes it possible for context-specific verification [52]. Further elucidation of ncRNA regulation for each subtype includes PseudoFun for pseudogenes [53] and GDCRNATools for lncRNAs [54]. The combination of HITS-CLIP/PAR-CLIP with subcellular RNA imaging [5] and mass spectrometry-based RBP abundance measurement allows analysis of sublocalization and RBP binding [6]. Taken together, the Smart Cancer Survival Predictive System and the Gene Survival Analysis Screen System are brought up for individual prognostic evaluation and precise clinical supervision [55].

## 5. Molecular Bases for ceRNA Interaction

Mathematical, in silico, and laboratory approaches have been carried out as above enumerated, yielding conclusions that abundance of ceRNAs and miRNAs, subcellular localization, the number of shared MREs, and many other indirect contributing factors are suggested to influence ceRNA-miRNA interaction efficiency.

Analyses for optimal cross-talk conditions showed that only when the stoichiometry of the interrelated ceRNA and miRNA falls in a narrow range of equivalence could significant cross-regulation occur [18, 56, 57]. Such swift mutual effects are initiated with a threshold-like behavior, accounting for miRNAs presenting both their roles as “switches” when target transcripts are highly repressed in low abundance and “fine-tuners” when ceRNA levels are floating around the threshold for sensitive regulation [58, 59], partly consistent with the assumption that higher amount of miRNAs for target ceRNAs exert stronger repressive effects [60]. Furthermore, with MREs in equal affinity for the shared miRNAs, the wider repertoire of ceRNAs targeted by the miRNA is, the weaker influence the miRNA would exert on each individual target [57]. In other words, distant ceRNAs in the same regulatory network bidirectionally detriment miRNAs' efficacy on each other. Notably, a quantitative assay for miR-122 and its ceRNA aldolase mRNA revealed that significant derepression for ceRNA rivals was only observed when aldolase mRNA experienced nonphysiological overexpression [59], indicating particular ceRNA crosstalk may be quite mild in normal conditions but prevalent in pathological contexts.

RNA-binding proteins (RBPs) are greatly involved in post-transcriptional regulation by means of RNA splicing, transport, and stability mediation. Except for MREs, there are RBP binding sites located in ceRNA sequences, whereby RBPs antagonize or cooperate with miRNAs by directly occupying specific binding sites or indirectly altering affinity to miRNAs through reordering the secondary structure of ceRNAs. With numerous binding locus neighboring or overlapping miRISC binding sites in 3′ UTR [61], RBP HuR largely stabilizes RNA transcripts, whereas AUF1 exerts synergistic effects with miRNAs on target energy [62]. Similarly, HuR recruits let-7 RISC for repression of transcriptional regulator c-Myc [63], and c-Myc is widely accepted in controlling miRNA transcription, including upregulation of oncogenetic miR-17-92 cluster [64]. Expectedly, RNAs may be relocated into different subcellular distributions once loaded with RBPs, which also affects the efficacy of spatiotemporally mutual interaction.

Hydrolytic deamination of adenosine to inosine (A to I editing), most frequently existing in UTRs and intronic sequences [65], epitomizes widespread RNA editing events in post-transcriptional regulation. It has been validated predominant in the majority of pre-mRNAs relying on adenosine deaminase, and to create new seed regions and accordingly corresponding target spectrum for miRNAs, destroying or generating miRNA matching substrates in ceRNAs [66]. Other forms of RNA editing resulting in base insertion, deletion, and nucleotide substitution simultaneously enrich the variation and diversity of the ceRNA network.

ceRNA crosstalk embraces multilayered regulatory hallmarks. Aside from the aforementioned aspects, the abundance of argonaute also causes competition among miRNAs as a bottleneck in the enzyme catalyzing process of miRISC synthesis [67]. Single-nucleotide polymorphism (SNP) allows subtle nucleotide component differences in MREs sharing collective miRNA pools, and alternative splicing provides miRNAs with shortened 3′ UTRs in a variety of cancer cells [68], both embodied in the altered affinity of MREs for miRNA binding, with higher affinity possessing stronger binding capacity. Hence, the overall regulating network is presented in given conditions, where additional determinants are exactly taken into consideration.

## 6. ceRNAs in Cancer

Chromosomal rearrangement, point mutation, shortened 3′ UTR, and other alterations in chromosome structure are commonly seen in the cancer cell genome, as a consequence, dysregulation of the ceRNA network and closely linked tumorigenesis, cell proliferation, and resistance to conventional treatment occur in such circumstances (Table 1).

As has been well elucidated, a decreased level of pseudogene PTENP1 leads to inhibition of tumor suppressor PTEN in a miRNA-dependent manner in numerous cancer types. The antisense lncRNAs, asRNAa, and asRNAb, derived from PTENP1 locus, respectively, recruit epigenetic regulators to the PTEN promoter region, confining PTEN transcription, and stabilize PTENP1 to derepress PTEN from miRNA absorption [69]. Coincident with previously published materials, ceRNA rivals for PTEN mRNA also includes mRNAs, whose competition for a large number of miRNAs is weakened in given pathological conditions, thus disrupting the downstream anti-oncogenic PTEN/AKT/*p*53 pathway [70].

Recent evidence deepens the body of knowledge concerning circRNAs in cancer progression. The absence of NUDT21, an RNA splicing factor, causes downregulation of circRNAs in HCC occurrence [71], and UGUA elements were pointed out to be crucial for sequence cyclization through binding with NUDT21 to form a dimer. Microarray revealed cirr5615 as an effective sponge for miR-149-5p, and its upregulation results in worse clinical outcomes in CRC patients, through disinhibition of *β*-catenin stabilization regulator tankyrase (TNKS) [72]. Similar mechanisms and positive correlation are found between increased circTP63 and FOXM1 levels in lung cancer, linked by miR-873-3p [73]. A prognostic model of N stage in TNM classification and overexpression of circCRIM1 was established based on circCRIM1-miR-422a-FOXQ1 crosstalk in nasopharyngeal carcinoma, which is related to peripheral implantation, epithelial-mesenchymal transition (EMT), and impaired chemosensitivity [74].

Constantly emerging lncRNA protagonists also provide new avenues for refinement of deregulated ceRNA interplay in carcinoma development. Based on vast achievements in this field, integrated analysis has risen up luxuriantly, delving into tissue- and cancer-specific differentially expressed genes (DEGs), harnessing intensive databases such as the Cancer Genome Atlas (TCGA) and the Gene Expression Omnibus (GEO), with statistical methods supporting bioinformatics analysis. Profile of lncRNA transcription has been outlined in a multitude of cancers such as HCC, breast cancer, glioblastoma, GC, and metastatic melanoma [75–80], providing promising biomarkers for prognostic evaluation and early-stage detection of these pathological changes.

Countless breakthrough in ceRNAs has undoubtedly sparkled diagnostic and curative potent towards cancer; what deserves extra attention, on the other hand, is that sometimes, it is the part of the integrated regulatory axis that paves the way for more outward-extending investigations. It has been revealed in 2018 that scaffold protein disabled-2 (DAB2), whose antineoplastic role was initially identified in ovarian cancer, was downregulated by miR-191 through binding with its MREs in 3′ UTR in response to estrogen stimulation, heralding promoted cellular viability, tumor growth, and poor long-term survival in patients with ER + breast cancer [81]. At the same time, the miR-203/SNAI2 axis emerged as a high-profile symbol in tumor stemness, EMT, and angiogenesis in prostate cancer, in which suppression of miR-203 on transcriptional inhibitor SNAI2 is relieved due to lessened miR-203 existence, rendering reanimation of the downstream oncogenic GSK-3*β*/*β*-CATENIN signal pathway by activated SNAI2 [82]. Later in 2019, similar molecular activities, somewhat replenishing the former, were unveiled in tumorigenesis of lung adenocarcinoma (LUAD), where overexpressed lncRNA chromatin-associated RNA 10 (CAR10) and lncRNA histocompatibility leukocyte antigen complex P5 (HCP5), which was transcriptionally up-regulated by SMAD3 after TGF*β* communication in advanced stages of LUAD, were both identified to prompt cell proliferation and metastasis exactly by means of miR-203/SNAI regulatory access [83, 84]. Such findings suggest a vast potential for future development with previous research.

## 7. Resistance to Immunotherapy and Chemotherapy

Despite endlessly upgraded triumphs in typical immunotherapies, such as monoclonal antibodies, immune checkpoint (IC) inhibitors, chimeric antigen receptor (CAR) genetically-modified T cell therapy, and genetic modification of T cell receptor (TCR), which are categorized into adoptive T cell therapy (ACT), tumor cells obstinately escape from internal or exogenously administered immune surveillance, through intrinsic, adaptive, or acquired accommodation, leading to malignant performance and nonresponse to immunotherapy. Cytotoxic T-lymphocyte-associated protein 4 (CTLA4) on T cells has been well studied as an immune checkpoint, which blocks T-cell-stimulating binding of CD28 and APC B7 molecules, inducing T cell inactivation [85]. Contrary regulatory trends of miR-29 and B7–H3, one of the eight isoforms of the B7 family, were confirmed in solid tumors such as neuroblastoma, sarcoma, cutaneous melanoma, and breast cancer [86–88]. Another commonly upregulated IC in cancers is PD-1, whose binding with its ligand PD-L1 induces T cell disability. Somatic mutation of guanine to cytosine in PD-L1 3′ UTR leads to altered MRE sequences in various gastrointestinal cancers (GCs), freeing PD-L1 mRNA from the restriction of miR-570 [89, 90]. Additionally, an array of miRNAs have been found related to aberrant overexpression of PD-L1 in both solid and hematological cancers and to motivate chemoresistance and metastasis. lncRNA-miRNA-PD-L1 regulations are also broadly discovered in tumor growth, cell proliferation, and migration in GCs and nonsmall cell lung carcinoma (NSCLC) [91–94]. Possible explanations for resistance to IC inhibitor (ICI) immunotherapy reside in the heterogeneity of MHC loss and susceptibility to spontaneous mutations, with deteriorated recognition and immune response to abnormal stimuli.

The development of TCR-engineered T cell therapy and CAR-T cell therapy reflects and, to a great extent, stands for the longstanding exploration of adoptive T cell therapy. Granted as a breakthrough designation with CD19-targeting CAR-T cell therapy on CD19+ B cell hematological malignancies leading to complete or partial remission in clinical trials, cancer immunotherapy focuses on fully arousing or assisting the autologous immune system to exert intensified supervision and restriction on tumor progression, but in fact, immune evasion is always inevitable ascribed to the ever-changing tumor microenvironment, immunosuppressive cytokine pathways (e.g., IFN*γ* in PD-L1 expression, TGF*β* in urothelial cancer, VEGF in producing myeloid-derived suppressor cells (MDSCs), Wnt/*β*-catenin signals in colorectal cancer, and lack of sensitizing cytokines such as IL-2, IL-12, and IL-15), impaired function or expression of antigen loading molecules and abnormal post-transcriptional background of ceRNAs, letting tumor cells subtly get away with immunological monitoring [95–100]. LncRNA MALAT1 drives dendritic cells (DCs) into tolerogenic types with the secretion of IL-10 and low-level expression of CD80 and MHC by acting as an miR-155 sponge [101]. Meanwhile, PD-L1 is overexpressed in the regulation of miR-195 on MALAT1 in diffuse large B-cell lymphoma and Linc00473 in pancreatic cancer [91, 102]. The above-mentioned lncRNA HOTAIR frees human leukocyte antigen-G (HLA-G) expression from miR-152, oppressing immune response through devitalizing NK cell activity in gastric cancer [103, 104]. CD8+ T cell fatigue and exhaustion, which is positively related with T cell immunoglobulin and mucin domain protein 3 (Tim-3), is a common route of immune escape in hepatic cell carcinoma and could be restored by inhibition of nuclear-enriched autosomal transcript 1 (NEAT1) to enhance Tim-3 capture via miR-155 [94]. Also, NEAT1 directly binds with DNMT1, a member of DNA methyltransferase family, with aberrant methylation in promoter regions leading to epigenetically downregulated antioncogene *P*53 expression and cGAS/STING pathway for T cell invigoration [105]. Here, we have merely touched on limited elements of ceRNA regulation in adoptive immunotherapy, while the understanding of therapeutic agents and regulatory hinges informs a feasible combination of T cell therapy with selected chemokines, cytokines, ICIs, and monoclonal antibodies, foreboding accessible application and benefits of optional strategies in cancer treatment.

Chemoresistance is an Achilles heel for progression and unsatisfying prognosis of malignancy and is always accompanied by disadjust biological mechanisms including drug outflow, cell proliferation, distant migration, and EMT. One of the most exploited regulators in chemoresistance is lncPVT1-representing noncoding sequences transcribed from the cancer-prone 8q24 chromosome [106] (Table 2). lncPVT1 is empowered with three regulating approaches. First, it recruits modifiers such as EZH2 to epigenetically dampen tumor suppressors, including *p*53 in HCC [107], large tumor suppressor kinase 2 (LATS2) in NSCLC [108], and miR-195 [109]. Second, differential processing of lncPVT1 is referred to the generation of lncPVT1-derived miRNAs, taking miR-1204 in NSCLC for instance, which accelerates cell proliferation through targeting paired-like homeodomain 1 (PITX1) [110]. Finally and predominantly, lncPVT1 dysregulation in resistance to chemotherapeutic agents towards a myriad of cancers reconciles its tremendous vitality as a ceRNA.

## 8. Conclusion

In this review, we present a genome-wide molecular interaction dominated by ncRNAs in a deep-going extent of post-transcriptional regulation regarding different types of cancer, where detailed mechanisms of the dynamic network, powerful predicting tools, and typical ceRNA crosstalk in pathogenesis, progression, and drug resistance in cancer have all together sketched out an encouraging blueprint for in-depth scientific research and translation into clinical application. Still, we have to acknowledge that despite extensive efforts endeavored into broadening our understanding of this realm, there is much more Terra incognita remaining to be carved out. Primarily, with most of the current research carried out on the overall cell-cluster level, intratumor heterogeneity among cancer cells has long been neglected, which is also crucial for neoplasia and therapeutic resistance. Here, we lay emphasis on two points of concern. First, even if numerous regulating nodes have been implicated as prospective targets for clinical therapy, accurate manipulation on these hubs without the involvement of other irrelevant locus waits for delicate Polish. Given that each single molecular tends to be the junction of, or to be indirectly covered by separate regulatory pathways with synergistic, antagonistic, or unrelated functions, controllable and unidirectional interventions would furthest avoid adverse reactions and achieve desired outcomes. Second, phenotypes observed through lowered expression or overexpression of single ceRNA/miRNA axis may need to be dialectically viewed, as its significance could be overmuch exaggerated under counteraction of other seemingly nonessential issues when conducting the research, or it is so tightly dragged by many other unclarified interlinks that enforced changes on the target axis alone is too weak to stand out in physiological conditions. There is no denying that booming advance in ceRNA network provides an exciting starting point for clinical practice and future research in cancer.

## Figures and Tables

**Table 1:** ceRNAs in cancers.

Cancer	ceRNA	miRNA	Target	Reference
Breast cancer	CYP4Z2P-3′ UTR	miR-211, miR-197, miR-204	CYP4Z1	[111]
	FOXO1 3′ UTR	miR-9	E-cadherin	[112]
	VERSICAN 3′ UTR	miR-136, miR-199a-3p, miR-144	Rb1, PTEN	[46]
	lncRNA GAS5	miR-21	—	[113]
	linc-ROR	miR-205	ZEB2	[114]
		miR-145	ARF6	[115]
	lncRNA–CDC6	miR-215	CDC6	[116]
CC	lncRNA XLOC_006390	miR-331-3p, miR-338-3p	PKM2, EYA2	[117]
CRC	OCT4B mRNA	miR-145, miR-20a/b, miR-106a/b, miR-335	OCT4A	[118]
	circ-ITCH	miR-7, miR-17, miR-214	ITCH	[119]
	circ5615	miR-149-5p	TNKS	[72]
Endometrial cancer	linc-ROR	miR-145	—	[120]
GC	lncRNA GAPLINC	miR-211-3p	CD44	[121]
	lncRNA HOTAIR	miR-331-3p	HER2	[122]
	LncRNA MT1JP	miR-92a-3p	FBXW7	[123]
HCC	lncRNA CCAT1	let-7	HMGA2, c-Myc	[124]
	lncRNA HOTTIP	miR-125b	—	[125]
	lncRNA HULC	miR-372	PRKACB	[37]
	LINC00974	miR-642	KRT19	[126]
	lncRNA UCA1	miR-216b	FGFR1	[127]
	Pseudogene INTS6P1	miR-17-5p	INTS6	[128]
	PTENP1	miR-17, miR-19b, miR-20a	PTEN,	[129]
	lncRNA FAL1	miR-1236	AFP, ZEB1	[130]
Lung cancer	lncRMA LCAT1	miR-4715-5p	RAC1	[131]
LUSC	circTP63	miR-873-3p	FOXM1	[73]
NPC	circCRIM1	miR-422a	FOXQ1	[74]
	lncRNA FAM225A	miR-590-3p, miR-1275	ITGB3	[132]
	lncRNA PTPRG-AS1	miR-194-3p	PRC1	[133]
	lncRNA ZFAS1	miR-892b	LPAR1	[134]
NSCLC	AEG-1 3′ UTR	miR-30a	Vimentin, snail	[135]
OC	lnc-OC1	miR-34a, miR-34c	—	[136]
Prostate cancer	CNOT6L/VAPA	miR-17, miR-19a, miR-20a/b, miR-106a/b, miR-93	PTEN	[25]
	lncRNA PCAT1	miR-3667-3p	c-Myc	[137]
	K-RAS1P	—	K-RAS	[24]
	PTENP1	miR-17, miR-19, miR-21, miR-26, miR-214	PTEN	[24]
	lncRNA UCA1	miR143	MYO6	[138]

Abbreviation: CC: cervical cancer; CRC: colorectal cancer; GC: gastrointestinal cancer; HCC: hepatocellular carcinoma; LUSC: lung squamous cell carcinoma; NPC: nasopharyngeal carcinoma; NSCLC: nonsmall cell lung cancer. —: not available.

**Table 2:** ceRNAs in chemotherapy resistance.

ceRNA	miRNA	Target	Chemotherapeutic agent	Cancer	Reference
LncPVT1	miR-195	SMAD3	Paclitaxel	CC	[139]
	miR-216b	Beclin-1	Cisplatin	NSCLC	[140]
	miR-152	c-MET	Gemcitabine	Osteosarcoma	[141]
	miR-1207-5p/3p	SMAD	Gemcitabine	PC	[142]
	miR-143	Hexokinase 2	—	GBC	[143]
	miR-186-5p	YAP1	—	HCC	[144]
lncRNA HOTAIR	miR-17-5p	Beclin1	Sunitinib	Renal cancer	[145]
lncRNA HOXA-AS2	miR-520c-3p	S100A4	Adriamycin	AML	[146]
lncRNA GAS5	miR-221	—	Gemcitabine	PC	[147]

Abbreviation: CC: cervical cancer; NSCLC: nonsmall cell lung cancer; PC: pancreatic cancer; GBC: gallbladder cancer; HCC: hepatocellular carcinoma; AML: acute myelocytic leukemia. —: not available.

## Data Availability

References in this review were mainly retrieved from PubMed, ClinicalKey, and OVID and were downloaded from respective websites (https://pubmed.ncbi.nlm.nih.gov/, http://www.clinicalkey.com, and http://ovidsp.ovid.com/) and http://www.yz365.com/.
